# Study of *FOXL2* Regulation on Ovarian Function in *Chlamys farreri* Through Comparative ChIP-Seq and Transcriptome Analysis Using RNA Interference

**DOI:** 10.3390/biology14091259

**Published:** 2025-09-12

**Authors:** Xiaoling Liu, Han Yun, Yan Xing, Shuo Wang, Xueying Zhou, Jianbai Zhang

**Affiliations:** 1 College of life Science, Yantai University, Yantai 264006, China; 2 Marine Economic Research Institute, Yantai 264006, China

**Keywords:** scallop, gender or gonadal development

## Abstract

*FOXL2* (forkhead box protein L2) is a transcription factor, its function and regulatory mechanism have been mainly studied in mammals; related research on marine invertebrates is still insufficient. It was found that oogenesis was affected, and even a small number of cells resembling spermatogonial morphology appeared in *C. farreri* ovaries after the *FOXL2* was knocked down through RNA interference (RNAi) technology in our laboratory previously. Based on previous research, this paper conducted transcriptome sequencing and differential expression analysis on the ovarian tissues between the experimental group (post-RNAi) and the control group (pre-RNAi) of *C. farreri*, and used recombinant *C. farreri FOXL2* protein for antibody production in Chromatin Immunoprecipitation Sequencing (ChIP seq) experiments to comprehensively analyze the pathways and key genes regulated by *FOXL2* during oogenesis. The results showed that in the RNAi experimental group, 389 genes were upregulated, and 1615 genes were downregulated. Among the differentially expressed genes (DEGs), the differential genes related to gender or gonadal development are relatively concentrated in physiological processes such as steroid hormone synthesis, spermatogenesis, gonadal development, and ovarian function maintenance, as well as the FoxO and estrogen signaling pathways. Combining transcriptome and ChIP-seq data, it was found that there were some genes related to sex gonadal development among genes which were directly regulated by FOXL2, such as *Wnt4*, *SIRT1*, *HSD17B8*, *GABABR1*, *KRAS*, *NOTCH1*, *HSD11B1*, *cPLA2*, *ADCY9*, *IP3R1*, *PLCB4*, and *Wnt1*. This study lays the foundation for a deeper understanding of the FOXL2′s specific regulatory mechanism during oogenesis in scallops as a transcription factor.

## Simple Summary

This study is about *FOXL2* regulation on *Chlamys farreri*’s ovarian function. Through comparative ChIP-Seq and transcriptome analysis using RNAi, we found that the *FOXL2* gene in the ovary of the scallop can directly or indirectly regulate some genes to exert its transcription factor function, which are concentrated in physiological processes such as steroid hormone synthesis, spermatogenesis, gonadal development, and ovarian function maintenance.

## 1. Introduction

*FOXL2* (forkhead box L2) is a member of the FOX transcription factor family. Relevant research has mainly been conducted in mammals, and it is believed to play a role in ovarian development and function maintenance [1,2,3,4,5,6]. *FOXL2* is a key factor in follicular development; *FOXL2* exerts its function by regulating the expression of genes such as *Gdf9*, *StAR*, *cyp19*, *Sox9*, *Sf1*, *FST*, *SIRT1*, etc. [1,2,3,4,5,6]. In vertebrates, it is particularly considered that the most important regulatory mechanism pathway of *FOXL2* is to regulate the expression of the *CYP19* gene, which in turn regulates estrogen synthesis and affects ovarian development [7]. Kuo et al. found that *FOXL2* can inhibit *CYP19* transcription and prevent ovarian senilism in Chinese hamster ovaries or granulosa cell lines [8]. However, Pannetier et al. found that *FOXL2* can activate the promoter of *CYP19* in *Capra hircus* (*C. hircus*) [9], indicating that the action mode of the *FOXL2* gene may vary in different species [7,8,9,10].

Through a series of preliminary studies on the *FOXL2* gene of *C. farreri*, our laboratory has first discovered the gene gender dimorphism expression in invertebrates and inferred that this gene was related to gender and gonadal differentiation in *C. farreri* [11]. Since then, the study of this gene in invertebrate has received increasing attention from researchers. Ye and Ren found that the *FOXL2* gene in *Hyriopsis cumingii* is mainly expressed in the ovaries, suggesting that *FOXL2* may play an important role in the ovarian development of *H. cumingii* [12]. Tang et al. showed that the expression of *FOXL2* in the ovaries of white spotted dogfish (*Esox lucius*) is 12-fold higher than that in the testes, exhibiting significant gender dimorphism [13]. He et al. analyzed the *FOXL2* expression in different tissues of *Crassostrea hongkongensi* and found that it was expressed the highest in the gonads [14]. Ren et al. described that the *FOXL2* expression was much higher in the ovaries of Thai fighting fish (*Bettas plendes*) than that in the testes, with significant gender differences [15]. Researchers have also found the female-related expression of *FOXL2* in *Patinopecten yessonsis* and *Cyclina sinensis* [16,17].

With increasing attention on *FOXL2* in shellfish, research on its functions has gradually expanded. Liu et al. used RNAi technology to knock down *FOXL2* and found that the oocytes’ morphology was abnormal, the nucleus was condensed, and oogenesis was significantly inhibited in *C. farreri*. Thus, the study indicated that *FOXL2* plays an important role in oogenesis in scallops [18]. Ning et al. also knocked down the *FOXL2* in *Argopecten irradians* through RNAi, the researchers found that the testis development-related genes *Dmrt1*, *Sox7*, and *Sox9* were all upregulated significantly with the decrease in *FOXL2* expression, while the ovary development-related genes *Vg*, *HSD14*, and *GATA-1* were downregulated manifestly [19].

So far, research on the regulatory target genes of *FOXL2* has mainly focused on vertebrates, and new target genes are constantly being discovered, such as *CYP26b1*, *HSD17b3*, *cdkn1b*, etc. [20]. The action mode of this transcription factor *FOXL2* in invertebrates, especially marine invertebrates, is still unclear. It is worth mentioning that some researchers considered that *CYP19* only appears in chordates, and so far, no *CYP19* sequence has been reported in any mollusks. At present, the regulatory target genes and pathways of *FOXL2* in bivalves remain unclear, and whether it functions through the conserved CYP19 pathway in vertebrates is still unknown. The specific regulatory role and target genes of *FOXL2* in scallops may be a new unknown scientific research field; the study of *FOXL2* in scallops can provide an important molecular biology basis for the reproductive regulation mechanisms of shellfish and the optimization of aquaculture technology.

## 2. Materials and Methods

### 2.1. Animal Samples

One hundred healthy scallops at an early proliferative stage were bought from a local seafood market near Yantai University (Yantai, China) and were temporarily kept in filtered seawater at about 16 °C. During the incubation period, the seawater was aerated continuously and replaced every day; scallops were fed with a mixture of 2 × 10^8^ *Phaeodactylum trecornutum* and 2 × 10^7^ *Chlorella vulgaris* every day [21]. According to the experimental methods described by Liu et al. [18] (our previous laboratory research), dsRNA used in RNAi were synthesized, and RNAi experiments were performed after the scallop culture was stable. The specific target sequence of dsRNA includes 41 bp of *foxl2* 3′UTR and 357 bp of *foxl2* CDS 3′ end. In brief, the RNAi methods were the following: the scallops were divided into three groups, each containing 25 individuals: the blank control group (non-injected), negative control group (PBS-injected), and experimental group (dsRNA-injected) [18]. The experimental group and negative control group were injected once a week; microinjectors were used to inject multiple points from the adductor muscle of the scallop. The experimental group was injected with dsRNA (50 μg dsRNA dissolved in 100 μL PBS) each time, while the negative control group was injected with 100 μL PBS; the gonadal tissues were collected on the 5th day after the second injection, then stored at −80 °C [18]. The *FOXL2* antibody used in the ChIP-seq experiment were derived from previous laboratory research [22].

### 2.2. RNA-Seq and Transcriptome Differential Expression Analysis

**Figure 1:**
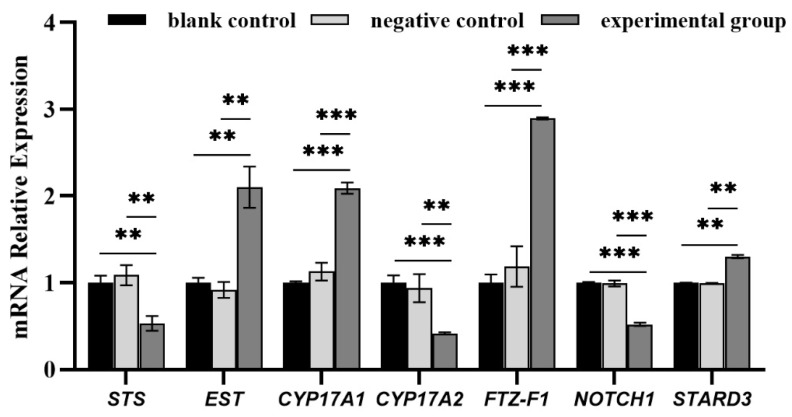
The mRNA expression of key DEGs. Note: * indicates significant difference (** *p* < 0.01, *** *p* < 0.001); the blank control group gene expression of each gene was, respectively, set as 1.00.

In this study, the non-injected group and PBS-injected group had no difference on the RNA expression of the genes (Figure 1), indicating that the injection did not affect the results. Total RNA extraction from each ovarian tissue was carried out using the guanidine isothiocyanate method [23], then the total RNA of three samples from the same group was mixed as one sample, then one pool sample from the negative control group and two pool samples (KD1/KD2) from the experimental groups (post-RNAi) were used for differentially expressed transcriptome sequencing. In particular, in order to improve the reliability of the research, the different parallel experimental groups KD1 and KD2 were set up. OE Biotech Co., Ltd. (Shanghai, China) conducted the Transcriptome-seq on the Illumina HiSeq X Ten platform.

Raw sequencing outputs (raw reads) underwent trimming and filtering via Trimmomatic and Trinity software (version: trinityrnaseq_r20131110) to yield clean reads. After the removal of adapter sequences and low-quality reads, these clean reads were initially assembled into expressed sequence tag clusters (contigs), and de novo assembled into transcripts using the paired-end method. Subsequently, paired-end sequencing methods facilitated the de novo assembly of these contigs into transcripts. For subsequent analyses, the longest transcript was selected as the Unigene based on transcript length and sequence homology. Bowtie2 software (version: 2.3.3.1) was employed to calculate Unigene expression, including both FPKM values [24] and read counts. To calculate the FDR value, the *p*-value is first computed for each Unigene and then adjusted for multiple hypothesis testing using the Benjamini–Hochberg (BH) method. Function annotations of differentially expressed genes (DEGs) were performed using Gene Ontology (GO) and Kyoto Encyclopedia of Genes and Genomes (KEGG) pathway analyses implemented in R software (version: 4.2.0) via hypergeometric distribution tests. RNA-seq data alignment using de novo transcriptome assembly was technically supported by OE Biotech Co., Ltd.

### 2.3. Real-Time Quantitative PCR Validation

Six DEGs pre- and post-RNAi, including *STS*, *EST*, *CYP17A1*, *CYP17A2*, *FTZ-F1*, *STARD3*, and *NOTCH1*, were verified using RT-qPCR on ABI 7500 Real-Time PCR System (Applied Biosystems, Foster City, CA, USA). RNA extraction used the same methods mentioned above; cDNAs were reverse-transcribed by reverse transcription kit (Beijing Takara Medical Technology Co., Ltd., Beijing, China). Primer sequences for target genes were detailed in Table 1. *β*-*actin* (GenBank accession: AY335441) served as the internal control. Three sample replicates per tissue and two technical replicates per sample were set up; the genes’ relative expressions were calculated using the 2^−ΔΔCT^ method, and significance was analyzed by SPSS software (version: 21) [25].

### 2.4. ChIP-Seq

The ChIP DNA was extracted using a Pierce Agarose ChIP Kit (Pierce Biotechnology, Rockford, IL, USA). Specifically, approximately 1 g of *C. farreri* ovaries were pulverized and cross-linked with 1% formaldehyde at room temperature for 10 min, then 1.1 mL Glycine Solution (10×) was added to terminate the cross-linking reaction, followed by washing twice with pre-cooled PBS (1×). Afterwards, the chromatin was extracted from the *C. farreri* ovarian nuclei, then fragments between 100 and 500 bp nuclease were obtained by microbial nucleases digestion (add 0.25 µL of Micrococcal Nuclease (MNase) (10 U/µL), shake well, and water bath at 37 °C for 15 min). In order to enrich the DNA fragments, which were bound to the target protein, 1–10 μL *FOXL2* antibody (pre-laboratory preparation, 21) was added to a 45 μL DNA fragment to form an antibody–target protein–DNA complex, and the yield was immunoprecipitated using Protein A/G Plus-Agarose. Following immunoprecipitation, complexes underwent washes with an elution buffer and were incubated overnight at 65 °C with 6 μL NaCl solution (concentration is 5 M) and 2 μL protease K solution (concentration is 20 mg/mL) to reverse the cross-linking. Then, the de-cross-linking product was purified and recovered. DNA was then purified and isolated. ChIP DNA sequencing was executed on a BGISEQ-500 sequencing platform, and raw images were processed into sequence data through base-calling and stored in FASTQ format. The resultant clean reads underwent alignment to the *C. farreri* genome [26] using SOAPaligner/SOAP2 software (version: 2.21t), and peak detection was performed using MACS software (version: 3.0) [26,27]. The entire ChIP-seq process was supported by BGI Genomics Co., Ltd. (Wuhan, China).

### 2.5. ChIP-qPCR Validation

The CHIP method for the *FOXL2* antibody experimental group, input group, and rabbit anti-IgG group is as described in Section 2.4 above, and the qPCR method is as described in Section 2.3 above, with the input group serving as the internal reference control. The relevant primers are shown in Table 1.

## 3. Results

### 3.1. RNA-Seq Analysis

#### 3.1.1. De Novo Assembly and Functional Gene Annotation

In all samples, the proportion of bases with a quality value greater than 30 (Q30) was over 92%, and the proportion of clean reads to the original data was over 90%, indicating good sequencing quality (Table 2).

**Figure 2:**
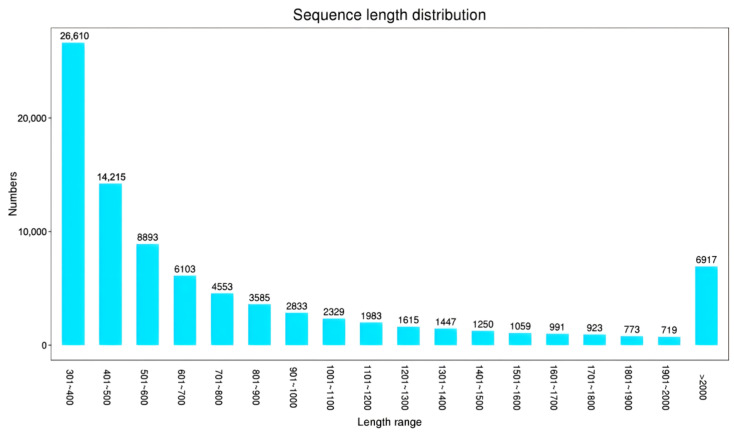
Unigene length distribution map.

All Unigenes were arranged in ascending order according to their sequence length (Figure 2). The sequence length is highest in the range of 301–400 bp, with 26,610 sequences, accounting for 30.7%. Subsequently, the overall distribution trend gradually decreases from 401 to 20.00 bp with increasing sequence length, with a total of 6917 large fragments longer than 20.00 bp, accounting for 8%.

#### 3.1.2. DEGs Analysis

**Figure 3:**
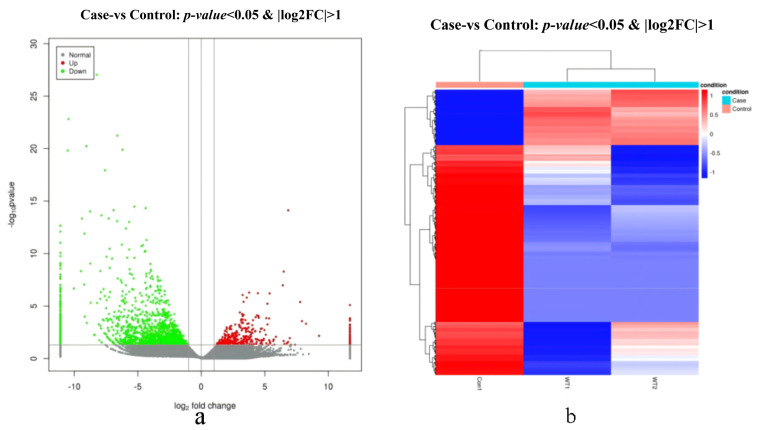
The volcano and heat maps of the DEGs. (**a**). Volcano plot of differentially expressed genes. Note: Red, green and gray represent significantly up—regulated genes, significantly down- regulated genes and non—significantly genes. (**b**). Cluster analysis of differential expression profiles. Note: Red indicates high expression genes and blue indicates low expression genes.

One differential subgroup was constructed to analyze DEGs using *p* < 0.05 and |log_2_FC| > 1. It was identified to have 2004 DEGs, containing 389 upregulated and 1615 downregulated genes. The volcano map (Figure 3a) and heat map (Figure 3b) of the DEGs are shown below.

#### 3.1.3. GO Annotation

**Figure 4:**
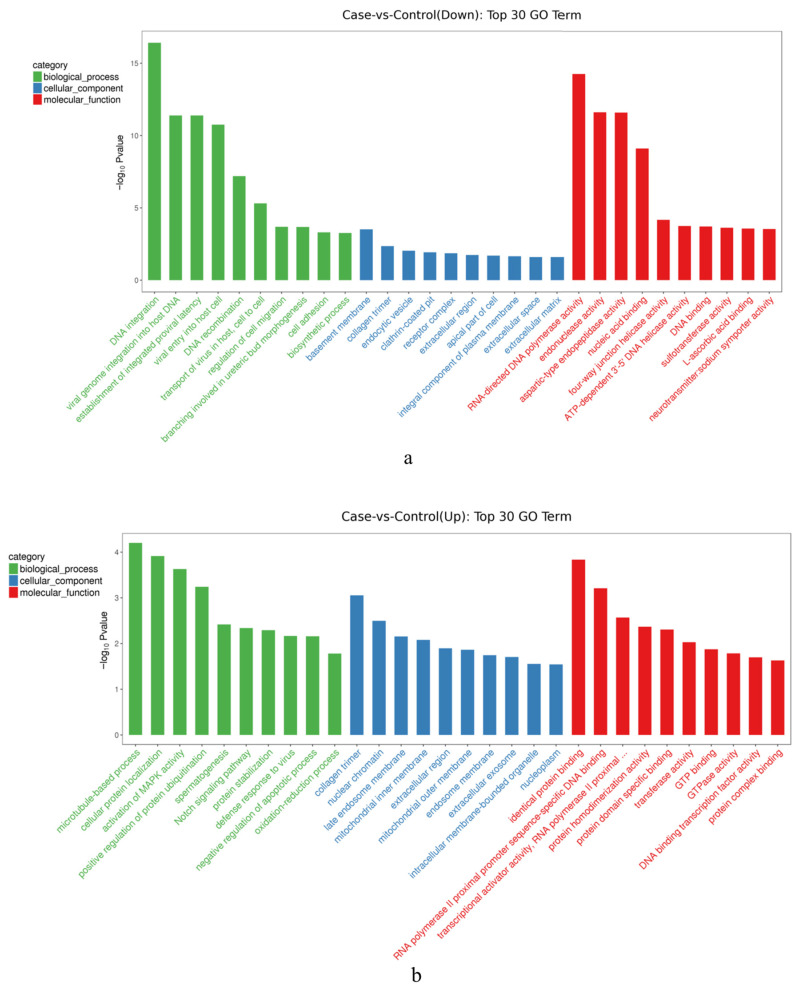
Up/downregulated DEGs GO enrichment top30 entries graph. (**a**). Down—regulated DEGs GO enrichment top30 entries graph; (**b**). Up—regulated DEGs GO enrichment top30 entries graph. Note: The Go categories are color-coded: green for biological process, blue for cellular component, and red for molecular function.

Downregulated DEGs (Figure 4a) were mainly enriched within biological processes such as DNA integration and viral genome integration into the host DNA. In terms of molecular function, downregulated DEGs were enriched basement membrane and collagen trimers. Most of the functions of upregulated DEGs (Figure 4b) were enriched within the collagen timer, nuclear chromatin, and late endosome membrane in the cellular component. In terms of molecular function, identical protein binding and RNA polymerase II promoter sequence-specific DNA binding contributed the largest proportion. With regard to biological processes, the cellular protein localization and spermatogenesis represented were the most prevalent.

Among the above DEGs related to gender gonadal differentiation were those mainly enriched in spermatogenesis. In addition to the top30, there were many other DEGs associated with sex determination and gonad differentiation mainly enriched in steroid hormone synthesis, gonadal development, and the maintenance of ovarian function.

#### 3.1.4. KEGG Annotation

**Figure 5:**
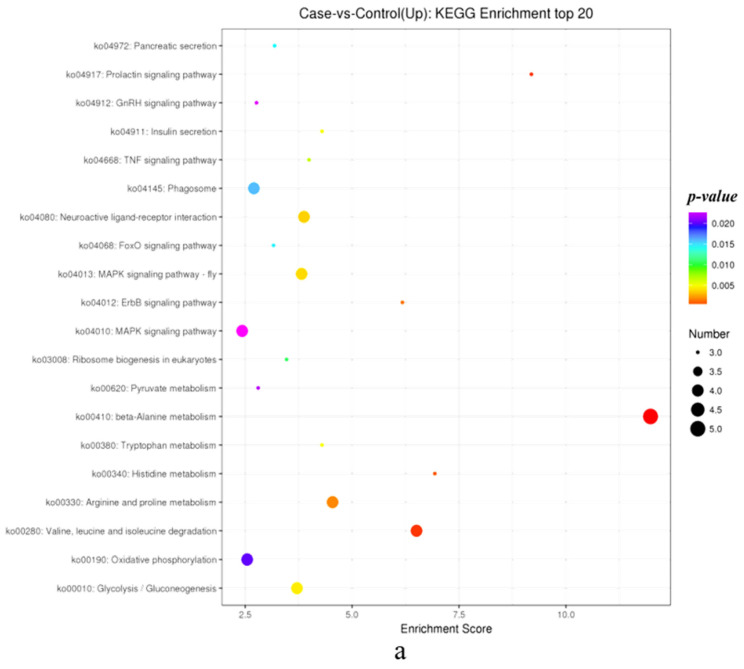

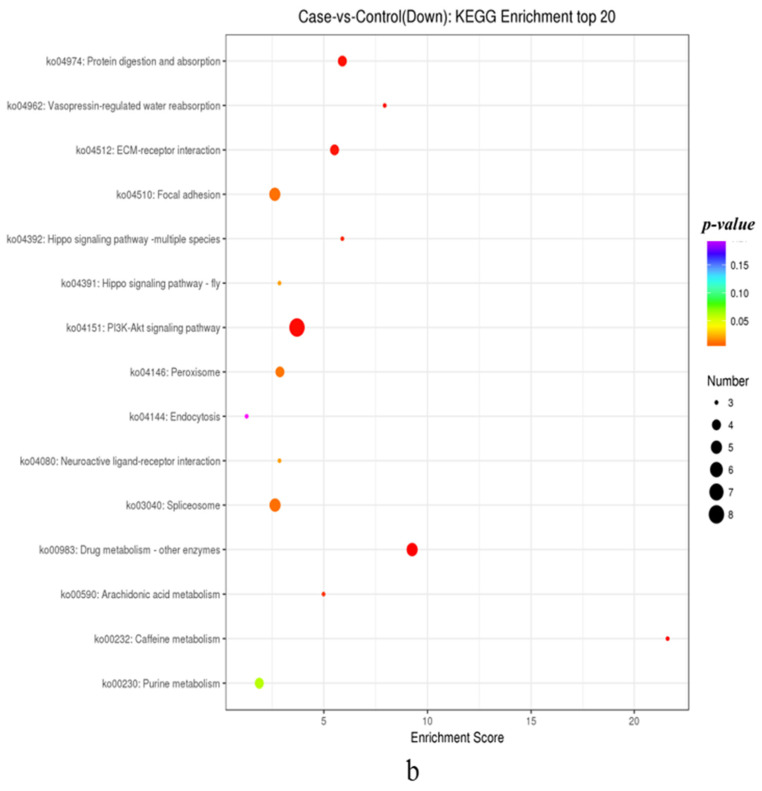
Up/downregulated DEGs KEGG enrichment top20 bubble map. (**a**). Up—regulated DEGs KEGG enrichment top20 bubble map; (**b**). Down—regulated DEGs KEGG enrichment top20 bubble map.

KEGG analysis showed that the MAPK signaling pathway, phagosome, and oxidative phosphorylation were the most enriched in upregulated DEGs (Figure 5a). The PI3k-Akt signaling pathway, protein digestion and absorption, and spliceosome were the most enriched in downregulated DEGs (Figure 5b). Among the top20 pathways, DEGs related to sex determination and gonad differentiation were mainly enriched in the FoxO signaling pathway. In addition to the top20 KEGG pathways, there were a large number of DEGs enriched in the Estrogen Pathway.

Combining the GO functional analysis of DEGs and KEGG signaling pathway analysis, genes related to steroid hormones, spermatogenesis, gonadal development, and oogenesis, as well as genes related to maintaining ovarian function and preventing premature ovarian failure, are all labeled in the caption of Figure 5.

Combined GO and KEGG analysis identified 20 genes associated with steroid hormones (*HSD17b8*, *StARD3*, *CYP3A*, *CYP2J*, *CYP20A*, *STS*, *EST*, *JUN*, *cPLA2*, *ADCY9*, *IP3R1*, *PLCB4*, *HSD11B1*, *CYP17A1*, *CYP17A2*, *ST1A1*, *SERK2*, *S5AR1*, *CaM*, and *Hras*), 13 genes associated with spermatogenesis were identified (*Dmrt1*, *Sox9*, *Nup62*, *GABABR1*, *BmHP21*, *Dhm1*, *LRRK2*, *DYNLL*, *ING2*, *PAIP-2*, *Tspan-8*, *LAMP-1*, and *CD107*), 6 genes related to gonadal development were identified (*FST*, *DAX-1*, *NOTCH1*, *Wnt4*, *MMP-17*, and *LRP-2*), 1 gene related to oogenesis (*MARF1*), and 1 gene related to the maintenance of ovarian function and the prevention of premature ovarian failure (*SIRT1*).

### 3.2. RT-qPCR Validation

Seven genes, *STS*, *EST*, *CYP17A1*, *CYP17A2*, *FTZ-F1*, *STARD3*, and *NOTCH1*, were verified for expression pre- and post-RNAi; the results (Figure 1) showed that the expressions of those genes were not significantly different between the negative and blank control groups, indicating that the factor of injection had little effect on the results of transcriptome sequencing. There were significant differences between the experimental group and the two control groups, indicating that experimental results were consistent with the trend of the transcriptome data, which verified the accuracy of the sequencing results.

### 3.3. ChIP-Seq Analysis

#### 3.3.1. ChIP-Seq Peak Analysis

The overview of the ChIP-Seq data of *FOXL2* is shown in Table 3, and the raw reads obtained from the two sequenced samples of FOXL2-IP and the input were the average of 23,747,152. The input was the control, which was the genomic nuclease-digested DNA. Without immunoprecipitation treatment, the DNA was directly delinked, purified, and analyzed. After quality filtration, 23,524,839 and 23,700,286 clean reads were obtained for FOXL2-IP and the input, respectively. Subsequently, the obtained clean reads were aligned to the *C. farreri* genome, and the mapped reads of FOXL2-IP and the input were 17,106,484 and 10,424,229, respectively.

**Figure 6:**
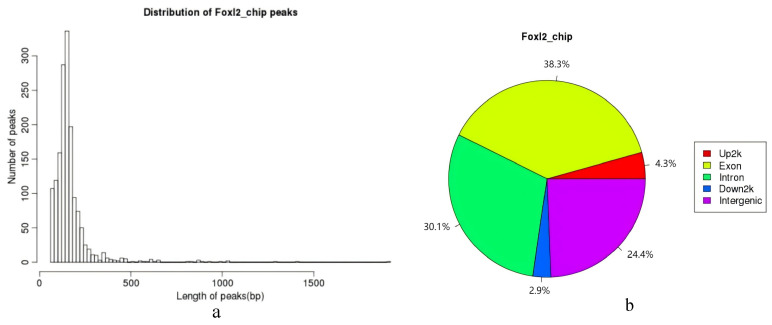
Length of peaks and peak distribution of gene elements. (**a**). Length of peaks of gene elements; (**b**). Peak distribution of gene elements.

A total of 1557 peaks were enriched and identified in the *C. farreri* ovary. The average length of the peak is 166 bp, and the peak width–length distribution is shown in Figure 6a, and the results show that most of the peak widths are around 200 bp. The distribution of the *FOXL2* target sequences on the gene functional elements was as follows: 24.4% in the intergenic region, 38.3% in the exon region, 30.1% in the intron region, 2.9% in the 3′-UTR end (Down2k), and 4.3% in the 5′-UTR end (Up2k) (Figure 6b).

#### 3.3.2. GO and KEGG Analysis of *FOXL2* Target Sequences

**Figure 7:**
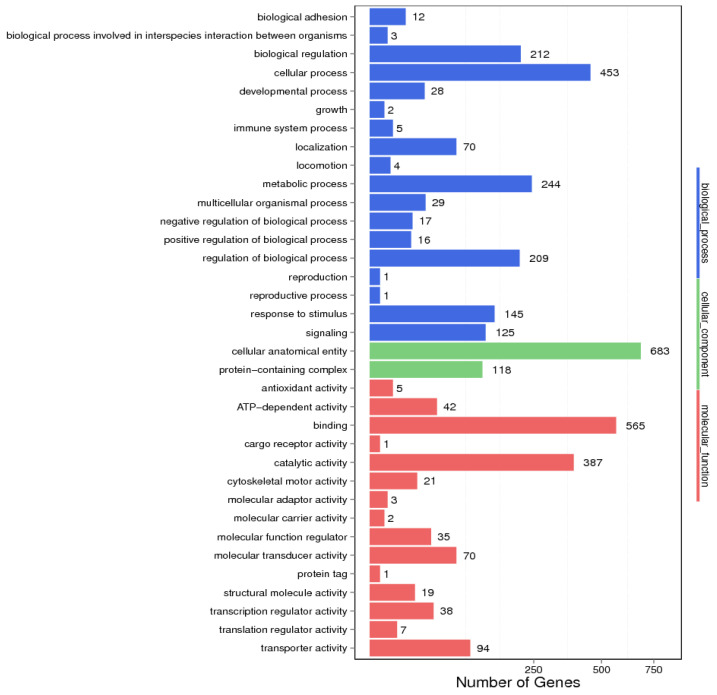
GO analysis of peak-related genes.

GO annotation analysis showed that the target sequences of *FOXL2* were mainly involved in biological processes such as the cellular process, reproduction, development, and metabolic process (Figure 7). In terms of cell components, they were mainly related to the cellular anatomical entity and protein-containing complex. For the molecular functions, the main functional annotations were involved in catalytic activity and transporter activity. The genes involved in the reproductive and developmental pathways related to the development of the gonads are mainly concentrated in the physiological processes of steroid hormone synthesis, gonadal development, and spermatogenesis.

**Figure 8:**
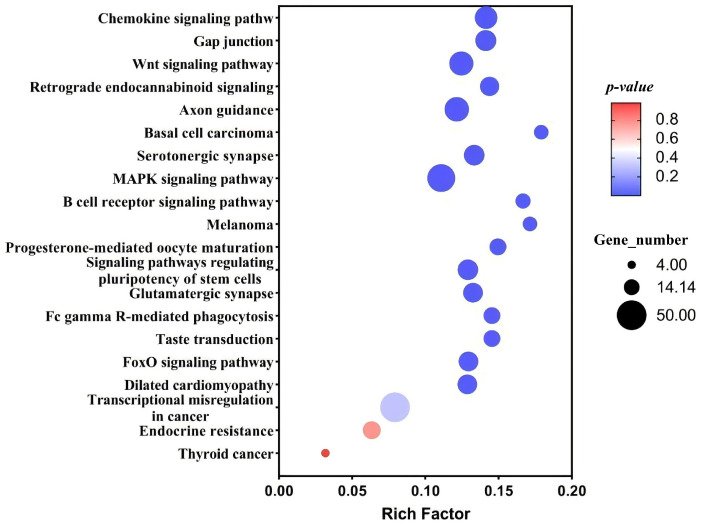
KEGG analysis of peak-related genes.

The KEGG pathway analysis provided insights into the metabolic pathways and the specific distribution of the *FOXL2* target sequences. The top20 metabolic pathways, as illustrated in Figure 8, revealed that the *FOXL2* target sequence was predominantly distributed in the chemokine, FoxO, and MAPK signaling pathways.

#### 3.3.3. Regulatory Candidate Genes of FOXL2

For the 1557 peak genes obtained above, the relevant information of these genes was analyzed by matching the *C. farreri* genome [26]. There were some genes related to sex gonadal development, as shown in Table 4, 16 steroid hormone-related genes, 2 spermatogenesis-related genes, 2 gonadal development-related genes, and 1 prevention of premature ovarian failure-related genes were identified. More detailed information for all the regulatory candidate genes is listed in Table 4.

#### 3.3.4. ChIP-qPCR

**Figure 9:**
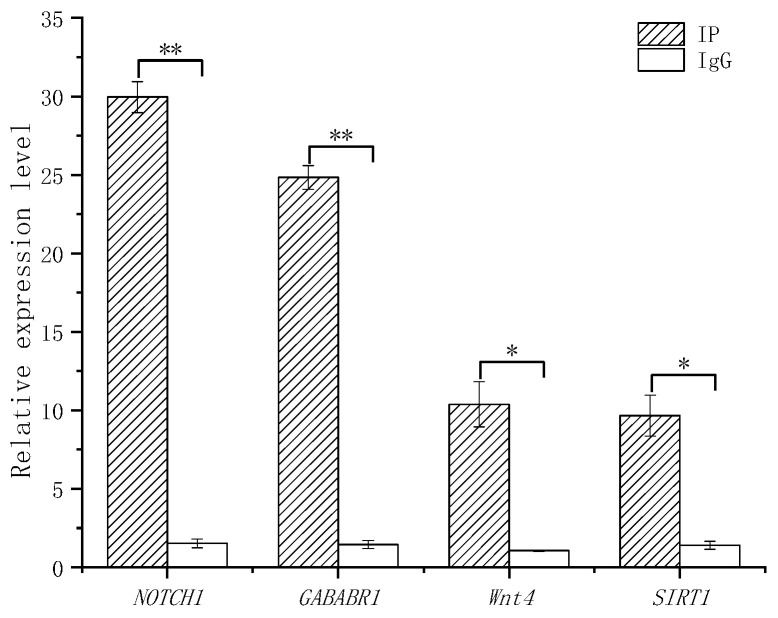
ChIP-qPCR analysis of four randomly selected target genes of *FOXL2.* Note: *: *p* < 0.05, **: *p* < 0.01.

Four randomly selected genes in Table 4 were validated using ChIP-qPCR, as shown in Figure 9; results showed that the expression level of the experimental group (IP) was significantly higher than that of the control group (IgG).

#### 3.3.5. Motif Analysis

**Figure 10:**
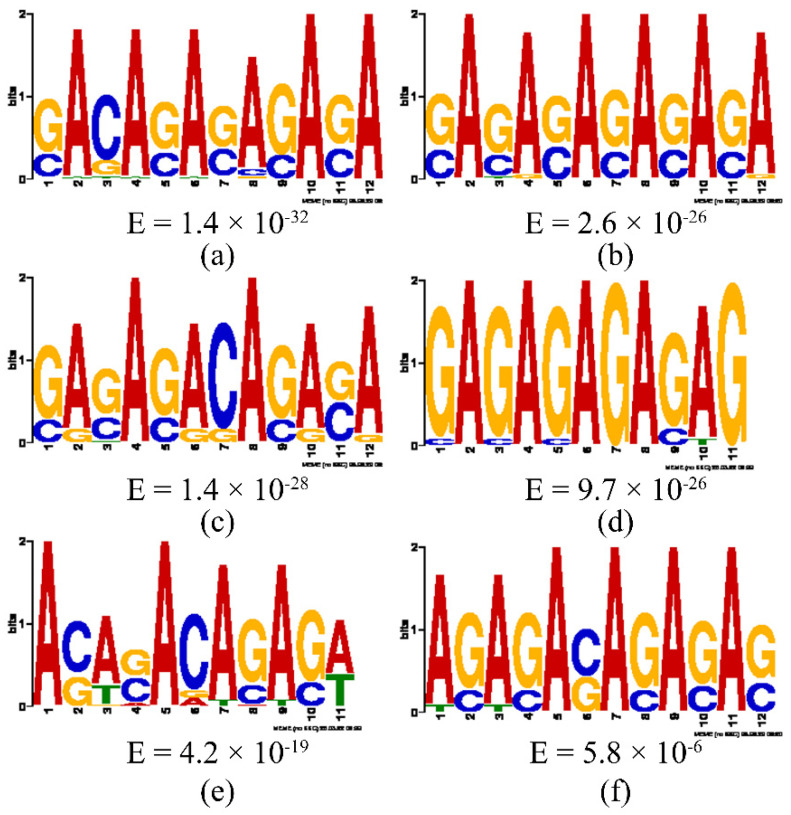
Motif analysis of peak sequences. (**a**) Motif 1; (**b**) Motif 2; (**c**) Motif 3; (**d**)Motif 4; (**e**) Motif 5; (**f**) Motif 6.

Based on the peak sequences, the following six motifs were predicted using the MEME software (version: 5.4.1) (Figure 10).

## 4. Discussion

Our laboratory has been studying the gonadal development of *C. farreri* for many years. Initially, we studied the expression of *FOXL2* in different tissues and found that *FOXL2* has significant ovarian expression specificity. We considered *FOXL2* to be a female-related gene and conducted a series of studies (e.g., we have studied the expression of *FOXL2* during gonadal differentiation, the expression pattern of the adult gonadal cycle, the expression pattern of embryonic development, and the function of FOXL2, etc.) [11,18,28,29].

The transcriptome differential expression showed 2004 DEGs, of which 389 genes were upregulated, and 1615 genes were downregulated; the number of downregulated genes was far greater than the upregulated genes, suggesting that *FOXL2* mainly upregulates genes’ expressions in scallop ovaries. This is consistent with the research on the regulation of *FOXL2* in chicken follicular granulosa cells [30].

Combining the transcriptome differential expression and ChIP *omics* analysis, it is considered that *FOXL2* can directly or indirectly regulate genes in multiple pathways to exert its role in the ovary. Firstly, target genes are included in the steroid hormone synthesis pathway, such as *HSD17B8StARD3*, *CYP3A*, *CYP2J*, *CYP20A*, *STS*, *EST*, *JUN*, *cPLA2*, *ADCY9*, *IP3R1*, *PLCB4*, *HSD11B1*, *CYP17A1*, *CYP17A2*, *ST1A1*, *KRAS*, *PGE2*, *YPEL1*, *SOS2*, *EFCAB11*, *AKT2*, and *SHC4*.

In invertebrates, little is known about the physiological sources of sex steroids, especially their biosynthetic pathways. Thitiphuree et al. state that *CYP17A*, *HSD3B*, *HSD17A*, *HSD17B8*, and *StAR3* genes are all important genes for steroid hormone synthesis in *Mizuhopecten yessoensis*. They suggest that *CYP17A* possesses both 17 α-hydroxylase and 17,20 lyase and is crucial for the production of sex steroids [31]. Previous research in our laboratory has found that there are two types of *CYP17* genes in *C. farreri*; *CYP17A1* was most expressed in the mature testis and growing ovary, suggesting that the gene may play a role in *C. farreri* testis development by participating in testosterone production and by also affecting oocyte growth. *CYP17A2* was expressed higher at the mature gonadal stage than at the other stages, suggesting a correlation with sex cell maturation or discharge [32]. Guo et al. also discovered two *CYP17A* genes in *Stronghlocentrotus intermedius*; the researchers suggested that *CYP17A1* plays an important role in the testis, and *CYP17A2* may play a role in the maturation process of oocytes by regulating the production of 17 α, 20 β-dihydroxy-4-pregnen-3-one (DHP, a progesterone) [33]. This paper analyzes the transcriptome differences pre- and post-*FOXL2* knockdown and suggests that *FOXL2* negatively regulates the expression of *CYP17A1* and positively regulates the expression of *CYP17A2*.

In mammals, *StAR* can promote the transport of cholesterol from the outer mitochondrial membrane to the inner mitochondrial membrane in steroid hormone synthesis cells, playing a key role in cholesterol metabolism and steroid hormone synthesis. So far, *StAR* genes have not been found in mollusks, but *StARD3* has been found to exist in some shellfish species [31,34]. *StARD3* was first found in the metastatic axillary lymph nodes of human breast cancer cells [35]; it has a START domain and belongs to the same subfamily as StAR in the START domain protein family. *StARD3* plays a role in the specific binding and targeting of cholesterol to specific organelle membranes and is involved in the synthesis of steroid hormones [36]. Studies have shown that STARD3, homologous to STARD1, can promote steroid hormone synthesis in mammalian tissues that do not express STARD1 [37,38]. Thitiphuree et al. detected *StAR3 (StARD3)* expression in *M. yessoensis* and speculated that *STARD3* in the steroid hormone synthesis pathway of *M. yessoensis*, like *StAR* in vertebrates, plays a role in transporting cholesterol to the mitochondrial inner membrane [31]. In our previous work, *StARD3* could also be found in *C. farreri*, and it was preliminarily speculated that *StARD3* may replace *StAR* in sex hormone synthesis [34]. In this paper, we found that *FOXL2* RNAi would upregulate *StARD3* expression (Figure 1), suggesting that *FOXL2* can inhibit *StARD3* expression, reduce progesterone synthesis, and prevent premature ovarian failure. After knocking down *FOXL2*, the expression of *STS* is downregulated, while the expression of *EST* is upregulated. It is considered that *FOXL2* positively regulates *STS* and negatively regulates *EST*, thereby affecting the activation or inactivation of estrogen production and participating in the regulation of steroid hormone balance in *C. farreri*.

Previous research in our laboratory has found that Fushi-tarazu factor-1 (*FTZ-F1*) is mainly expressed in testes at the mature stage, with a significantly higher expression than in the male and female gonads at other stages. It is speculated that *FTZ-F1* is involved in testosterone production to regulate testicular development in scallops [32]. In this paper, after knocking down *FOXL2*, the *FTZ-F1* expression was upregulated (Figure 1). It is considered that *FOXL2* negatively regulates *FTZ-F1*, affecting testosterone production in the ovaries and avoiding testicular development. This is consistent with the regulation of *Sf-1* (a subfamily of *FTZ-F1* genes in mammals) by *FOXL2* in mammals [39]. *CYP19* encodes aromatase, which catalyzes the conversion of testosterone to estradiol and is a key enzyme in the conversion of testosterone to estrogen. Data suggest that the *CYP19* gene appears after chordates and has not been found in mollusks [40]. Thitiphuree et al. suggest that there may be other aromatase genes replacing *CYP19* in invertebrates [31]. In this study, we found that the target genes of *FOXL2* contain multiple P450 family genes, such as *CYP3A*, *CYP2J*, *CYP20A*, etc., and there is a significant difference in expression pre- and post-*FOXL2* RNAi. Whether other aromatase genes replace *CYP19* in scallops still needs to be further researched and explored.

Shao et al. suggested that *GABAR* may be closely related to human sperm production [41]. Wang Yi et al. found that the expression of *GABABR* in the testes of gibel carp (*Carassius auratus gibelio*) was significantly higher than that in the ovaries, the researchers suggested that it may play an important role in testicular development or spermatogenesis [42]. Saito et al. found that male mice lacking *ING2* exhibited abnormal sperm production and infertility, indicating that *ING2* plays an important role in mammalian spermatogenesis [43]. In mammals, autophagy is a process that maintains cellular homeostasis and plays an important regulatory role in spermatogenesis [44]. Moreover, it was found that lysosome-associated membrane protein 1 (*LAMP1*) is involved in the autophagy of Chinese soft-shelled turtle (*Pelodiscus sinensis*) sperm [45]. In this research, we found that after *FOXL2* RNAi, the expression of genes related to spermatogenesis, such as *GABABR*, *ING2*, and *LAMP1*, were all upregulated. It is considered that *FOXL2* prevents spermatogenesis-related genes from differentiating and developing towards the testis by negatively regulating them.

In addition, we found that knocking down *FOXL2* downregulated the expression of *SIRT1*, indicating that *FOXL2* positively regulates the expression of this gene. A study has found that after *FOXL2* mutation in mice, the promotion of *SIRT1* is lost, accelerating follicular development and activating a large number of primordial follicles, leading to premature ovarian failure [6]. *SIRT1* is a key gene in the FoxO signaling pathway, which can directly or indirectly deacetylate FoxO to inhibit FoxO-mediated cell apoptosis and delay the aging process [46]. The positive regulation of *SIRT1* by *FOXL2* in *C. farreri* may be related to slowing down ovarian aging.

We found that the *FOXL2* target included genes related to ovarian development, as *FOXL2* can positively regulate *FST* expression, which in turn affects ovarian development. Ni et al. found that the expression of *FST* in the ovaries of *Crassostrea angulata* increases continuously with development and reaches its highest level at the mature stage, whereas the expression of *FST* in the testis remains at a low level throughout development and significantly decreases at the mature stage [47]. Therefore, it is considered that *FST* plays an important role in the development of oyster ovaries. Another author found that *Wnt4* has the highest expression level at the mature stage of the *C. farreri* testes and ovaries, and the expression level in the testes is significantly higher than in ovaries. It is speculated that *Wnt4* may be involved in the regulation of the development and maturation process of the gonads in both sexes, and its role in the testes is more significant than that in the ovaries [48]. On the other hand, it was found that the expression level of *DAX1* in the testes of *C. farreri* is significantly higher than in the ovaries, and *DAX1* is also highly expressed at the proliferative and growing stage of the ovaries. It is speculated that *DAX1* may be involved in the development of *C. farreri* testes and early ovaries. This study found that knocking down *FOXL2* upregulated the expression of *Wnt4* and *DAX-1*, suggesting that *FOXL2* has a negative regulatory effect on them. Previous research has found that *MARF1* can control meiosis in oocytes, and mutations in *MARF1* can lead to infertility in female mice because oocytes fail to undergo meiosis and release immature eggs, indicating that this gene plays an important role in the process of oogenesis [49,50]. This study found that after *FOXL2* RNAi, the *MARF1* expression was downregulated. It is believed that *FOXL2* positively regulates the expression of *MARF1* to assist in the normal development of ovum into mature ovum.

However, there is relatively little information on the binding sequence of target genes by *FOXL2*. Pisarska et al. found the sequence of the binding site between *FOXL2* and the target gene *StAR* in mice, which is similar to the motif discovered in this study [1]. In addition, through analysis of *FOXL2* binding sites, it was found that the vast majority of binding sites are located within genes, with 30.1% of binding sites in intronic regions and 38.3% in exon regions. This is similar to the study of *FOXL2* binding sites carried out by Barbara et al. in fetal ovaries [20]. Through the verification of CHIP-seq results, this study found that some binding sequences located inside genes (such as Figure 10e) have the characteristic sequences of enhancers. The function of intronic enhancers has not been explored to a large extent but has attracted more and more attention from researchers in recent years. The *FOXL2* binding sequence data suggest that transcriptional regulation may not exclusively focus on the promoter and its upstream regulatory region, as traditional research has done, and that the focus on introns and intron-type enhancers may also be important in regulatory studies.

## 5. Conclusions

In the process of oogenesis in *Chlamys farreri*, *FOXL2* mainly functions through the regulation of upregulated genes, and it can regulate genes in multiple pathways to exert its role in the ovary. This paper screened out some key target genes of *FOXL2* from the above pathways, laying an omics foundation for future, detailed research on the ovarian development mechanism of *Chlamys farreri*.

## Figures and Tables

**Table 1:** The sequences of the primers used in the experiment.

Gene Name	Primer Sequence (5′→3′)	Product Length	Melting Temperature	Usage
Steroid Sulfatase (*STS*)*-F*	GGGTTCTTTTGTTCGTCTGGC	146 bp	58.97	RT-qPCR
Steroid Sulfatase (*STS*)*-R*	TTTCCCGGGCTTCCAAGAATT		59.35	
Estrogen Sulfotransferase (*EST)-F*	GGTGTGATGAAGGTCAAGGG	105 bp	60.67	RT-qPCR
Estrogen Sulfotransferase (*EST)-R*	TACACATCGCCACAGAAGCA		59.78	
Cytochrome P450c17A1 (*CYP17A1*)*-F*	CCAAGTAGCCGATTCAAAAAAGTGT	144 bp	59.81	RT-qPCR
Cytochrome P450c17A1 (*CYP17A1*)*-R*	TCCAGCAAAGAAAATGTCAGCA		60.87	
Cytochrome P450c17A2 (*CYP17A2*)*-F*	GATGTGGACGATGCTTTTCTC	114 bp	60.03	RT-qPCR
Cytochrome P450c17A2 (*CYP17A2*)*-R*	TGTTTTGCCTGTTGCTGTTC		59.73	
Fushi-Tarazu Factor-1 (*FTZ-F1*)*-F*	TAGAGGCAGTGAGACAGGATAGAA	139 bp	59.65	RT-qPCR
Fushi-Tarazu Factor-1 (*FTZ-F1*)*-R* StAR-Related Lipid Transfer Protein 3 (*STARD3*)*-F* StAR-Related Lipid Transfer Protein 3 (*STARD3*)*-R*	GATTGCTGGGTCGGGTTTA GCGGGGTAGACAAACGGA ATGTCTCCTGTTCGCCGT	128 bp	60.78 60.54 60.02	RT-qPCR
Neurogenic Locus Notch Homolog Protein 1 (*NOTCH1*)*-F1*	GTTTACATCTGCTGAAGTGTGGAT	155 bp	59.59	RT-qPCR
Neurogenic Locus Notch Homolog Protein 1 (*NOTCH1*)*-R1*	CTCTGTGTCTTTTCTAGCCGTGTA		60.15	
*β-actin-F*	TTCTTGGGAATGGAATCTGC	119 bp	58.89	RT-qPCR
*β-actin-R*	GCCAGACTCGTCGTATTCCT		58.12	
Neurogenic Locus Notch Homolog Protein 1 (*NOTCH1*)*-F2*	GGAGAGGGACAACCAACACC	117 bp	58.7	ChIP-qPCR
Neurogenic Locus Notch Homolog Protein 1 (*NOTCH1*)*-R2*	TCACATTTGGATGGTTTCTGGA		60.2	
γ-Aminobutyric Acid Type B Receptor Subunit 1 (*GABABR1*)*-F*	ATCAAGTGGTCCGCAACTCT	49 bp	56.8	ChIP-qPCR
γ-Aminobutyric Acid Type B Receptor Subunit 1 *(GABABR1*)*-R*	TCATTGCATGACCTGTTGCC		59.7	
Wingless-Type MMTV Integration Site Family, Member 4 (*Wnt4*)*-F*	AGGTTGGGAAACCCTTGC	55 bp	57	ChIP-qPCR
Wingless-Type MMTV Integration Site Family, Member 4 (*Wnt4*)*-R*	CACAACTTGGCAGCACCA		56.1	
Silent Mating Typeinformation Regulation 2 Homolog 1 (*SIRT1*)-*F*	GCCAAGCAGTTCAACATCAA	74 bp	56.8	ChIP-qPCR
Silent Mating Typeinformation Regulation 2 Homolog 1 (*SIRT1*)-*R*	CTCCTGATGTTCCACAAATCC		56.7	

Note: The amplification efficiency of the genes in the table has reached around 95%.

**Table 2:** Sequencing data quality pre-processing results.

Sample	Raw_Reads	Clean_Reads	Valid_Reads (%)	Q30 (%)	GC (%)
Con1	49,024,148	45,647,374	89.53	92.31	40.81
KD1	44,400,934	42,162,166	91.97	93.62	41.92
KD2	49,725,368	46,588,672	90.16	92.68	40.45

**Table 3:** Statistical analysis of raw data.

Sample	Clean Reads	Clean Ratio	Mapped Reads	Mapped
FOXL2-IP	23,700,286	99.80%	10,424,229	43.98%
Input	23,524,839	99.06%	17,106,484	72.72%

Note: Clean reads: the number of reads obtained by filtering raw reads; mapped reads: the total number of reads on the alignment; and mapped rate: the proportion of the total number of reads on the alignment.

**Table 4:** Target genes of *FOXL2*.

Gene ID	Peak Start	Peak End	Peak Annotation	Gene Name
**Steroid Hormone**				
110441780	1,660,594	1,660,681	estradiol 17-beta-dehydrogenase 8-like	*HSD17b8*
110447098	2,005,851	2,005,913	cytochrome P450 2J6-like	*CYP2J6*
110451342	431,036	431,098	cytosolic phospholipase A2-like	*cPLA2*
110451344	520,879	520,970	prostaglandin G/H synthase 2-like	*PGE2*
110461839	815,032	815,194	adenylate cyclase type 9-like	*ADCY9*
110445220	505,918	506,027	protein yippee-like 1	*YPEL1*
110447714	109,981	110,076	son of sevenless homolog 2-like	*SOS2*
110445266	925,477	925,554	EF-hand calcium-binding domain-containing protein 11-like	*EFCAB11*
110459812	161,479	161,605	RAC-beta serine/threonine-protein kinase B-like	*AKT2*
110460076	25,292	25,362	SHC-transforming protein 4-like	*SHC4*
110460372	108,457	108,539	inositol 1,4,5-trisphosphate receptor type 1-like	*IP3R1*
110461441	1,140,080	1,140,262	1-phosphatidylinositol 4,5-bisphosphate 2-phosphodiesterase beta-4-like	*PLCB4*
110445501	1,702,410	1,702,520	GTPase HRas	*Hras1*
110463419	101,557	101,621	hydroxysteroid 11-beta-dehydrogenase 1-like protein	*HSD11B1*
110464786	243,337	243,491	lysosome membrane protein 2-like	*LAMP2*
110449851	223,863	224,009	neurogenic locus notch homolog protein 1-like	*NOTCH1*
**Spermatogenesis**				
110459375	1,049,938	1,050,020	gamma-aminobutyric acid type B receptor subunit 1-like	*GABABR1*
110456833	188,225	188,363	golgin subfamily A member 3-like	*GOLGA3*
**Gonadal Development**				
110451002	72,929	73,016	protein Wnt-4-like	*Wnt4*
110455012	1,822,037	1,822,131	insulin-like peptide receptor	*ILPR*
**Prevention of Premature Ovarian Failure**				
110460060	130,987	131,077	NAD-dependent protein deacetylase sirtuin-1-like	*SIRT1*

Note: The bold are different pathways of action related to sex gonadal development in *FOXL2* target genes.

## Data Availability

In the next few years, several graduate students in our laboratory will conduct further research based on these omics data. If the total data are leaked in advance, it will affect our laboratory follow-up research. So, based on the above considerations and the policies and confidentiality agreements adhered to in our laboratory, we cannot provide the total raw data. If anyone has questions about specific data, they can contact the corresponding author, and we will do our best to provide more detailed elaboration.
